# The effect of plyometric training and moderating variables on stretch-shortening cycle function and physical qualities in female post peak height velocity volleyball players

**DOI:** 10.3389/fphys.2024.1346624

**Published:** 2024-02-08

**Authors:** Richard Sylvester, Michal Lehnert, Ivana Hanzlíková, Jakub Krejčí

**Affiliations:** ^1^ Sport Performance Research Institute New Zealand, Auckland University of Technology, Auckland, New Zealand; ^2^ Department of Sport, Faculty of Physical Culture, Palacký University Olomouc, Olomouc, Czechia; ^3^ Department of Physiotherapy, Faculty of Physical Culture, Palacký University Olomouc, Olomouc, Czechia; ^4^ Department of Natural Sciences in Kinantropology, Faculty of Physical Culture, Palacký University Olomouc, Olomouc, Czechia

**Keywords:** girls, stiffness, maturation, plyometric exercises, reactive strength index, youth sport

## Abstract

**Purpose:** Although several studies investigated the effect of plyometric training on physical performance, there is a lack of clarity regarding the effectiveness of plyometric training or its moderator variables in youth female volleyball players. The primary aim of this study was to explore the effect of horizontal plyometric training on explosive stretch-shortening cycle hops and jumps in the vertical and horizontal directions in female post peak height velocity (PHV) volleyball players. The secondary aim was to assess the influence of participant and training related moderators on horizontal plyometric training in post-PHV volleyball players.

**Methods:** A total of 23 post-PHV volleyball players participated in this 8-week intervention with horizontal plyometric exercises, twice a week. Pre-testing and post-testing included bilateral and unilateral vertical sub-maximal hopping, horizontal jumping and hopping, and a drop jump test. The effectiveness of the intervention was assessed using a paired *t*-test. The influence of internal moderators such as age, maturity and body mass and external moderators such as training volume were assessed using regression and correlation analysis.

**Results:** An 8-week plyometric training improved sub-maximal hopping at 2.5 Hz left by 4.4%, bilateral sub-maximal hopping at 2.0 Hz by 9.5% and bilateral sub-maximal hopping at 2.2 Hz by 6.8% in post-PHV female volleyball players. Horizontal jumping and hopping, reactive strength index and other sub-maximal hopping conditions did not improve significantly. Body mass had a large moderating effect on vertical unilateral sub-maximal hopping at 2.5 Hz right (*p* = 0.010, *η* ^2^ = 0.314), vertical unilateral hopping at 3.0 Hz right (*p* = 0.035, *η* ^2^ = 0.170), and vertical unilateral hopping at 3.0 Hz left (*p* = 0.043, *η* ^2^ = 0.203). Training volume together with generalized joint hypermobility moderated right leg triple broad hop performance, whereas maturity and age did not moderate any variables.

**Conclusion:** This study determined that 8 weeks of horizontal plyometric training can improve unilateral absolute leg stiffness in post-PHV female volleyball players, and this training effect can be moderated by body mass. Furthermore, the training effect on triple hopping performance on the right leg can be moderated by combined training volume with generalized joint hypermobility.

## 1 Introduction

The stretch-shortening cycle (SSC) involves the coupling of a stretch action and rapid shortening action, divided by a very brief pause (Pedley et al., 2020). The stretch action primarily involves an eccentric muscle action and lengthening of the tendon in series, whereas the shortening action primarily involves a concentric muscle action and a shortening of the tendon in series (Turner and Jeffreys, 2010). The SSC plays an important role in jumping ability (Kums et al., 2005; Idrizovic et al., 2018), which is a fundamental component for offensive and defensive actions in volleyball (Giustino et al., 2022). These actions require a high level of explosive ability to successfully execute these jumps within the context of a match (Lehnert et al., 2017). Jumping high and jumping quickly are relevant skills to develop in volleyball players (Fatthi and Sadeghi, 2014; Rojano-Ortega et al., 2021), both during which effective SSC function encourages more efficient yielding and propulsive phases (Idrizovic et al., 2018; Carrasco-López et al., 2019). Plyometric training can be used to improve force and power output by improving SSC function (Kums et al., 2005; Carrasco-López et al., 2019). Effective SSC function optimizes elastic energy storage and return, increases stretch-reflex contribution, and increases neuromuscular recruitment and activation (Turner and Jeffreys, 2010; Radnor et al., 2017). These potentiating mechanisms thus lead to improved control and coordination of the yielding phase, reduced metabolic cost of movement, enhanced propulsive force, and greater force at a given velocity during the propulsive phase (Flanagan and Comyns, 2008; Turner and Jeffreys, 2010).

SSC function is governed by the effective interaction between neural, muscular and muscle-tendon structural factors (Radnor et al., 2017). The development of neural factors consists of improved recruitment and activation in the agonist muscles, improved stretch reflex function, and improved intermuscular coordination (Lambertz et al., 2003; Grosset et al., 2005; Grosset et al., 2008; Markovic and Mikulic, 2010; Asadi et al., 2017), while muscular factors include increased muscle size. Additionally, muscle-tendon factors include increased tendon size, and improved Young’s modulus, which is an indication of intrinsic material properties reflected by the stress-strain relationship (Waugh et al., 2012). Improvement in these factors may contribute to improved force production, increased rate of force production, improved stiffness, and overall SSC function, leading to improved jump and sprint performance (Radnor et al., 2017; Tumkur Anil Kumar, 2021).

The neuromuscular regulatory factors that govern the SSC develop as an individual grows and matures. Briefly, growth and maturation consist of the increase in size and progress towards a mature state of the body (Lloyd et al., 2014b; Radnor et al., 2021). These changes lead to a non-linear natural development of the SSC throughout childhood and adolescence (Radnor et al., 2017). To make improvements over and above the natural development of physical qualities, a developmentally appropriate training stimulus is required (Lloyd et al., 2016). More specifically, a training stimulus like plyometric training, targets the SSC and matches the natural adaptive processes, thus resulting in a synergistic relationship that leads to more effective adaptation (Lloyd et al., 2015; Moran et al., 2018). In youth, plyometric training enhances the ability to use the SSC, improving jump height, jump distance, reactive strength index (RSI) (Turner and Jeffreys, 2010; Johnson et al., 2011; Behm et al., 2017; Moran et al., 2018), stiffness (Lloyd et al., 2012a; Hammami et al., 2016), and power (Johnson et al., 2011; Lloyd et al., 2012a; Asadi et al., 2017).

The effectiveness of a plyometric training program is dependent on the training content and the external and internal moderator variables. External moderator variables include program variables like intensity, volume, program duration, total training sessions, and training frequency (Johnson et al., 2011; Asadi et al., 2017; Moran et al., 2018). For outcomes like countermovement jumping, longer duration interventions with higher frequency have led to a greater adaptive response (Moran et al., 2018), whereas for outcomes like stiffness, shorter durations of training have proven effective in boys, but are largely unknown in girls (Lloyd et al., 2012a; Ramirez-Campillo et al., 2023a). These findings suggest that plyometric training effectiveness may differ between girls and boys due to maturational related adaptations (Lloyd et al., 2015; Moran et al., 2018; Ramirez-Campillo et al., 2023a). The findings also suggest that other internal moderator variables such as age, maturity, body size and generalized joint hypermobility (GJH) are also important to consider (Radnor et al., 2017; Simmonds, 2022). However, there are fewer studies in girls that evaluate the effect of plyometric training only on leg stiffness, standing long jumps, broad hops (Myer et al., 2005), triple broad hops (Noyes and Barber-Westin, 2015), or RSI compared to boys (Moran et al., 2018; Bogdanis et al., 2019; Ramirez-Campillo et al., 2023b). Second, there are fewer studies in girls that evaluate the influence of external moderators like training volume, or internal moderators like chronological age and maturity timing, body size, body composition, and total GJH score (Moran et al., 2018; Ramirez-Campillo et al., 2023a). There is a limited understanding of plyometric training effectiveness in girls during both childhood and adolescence (Ford et al., 2011; Moran et al., 2018; Pichardo et al., 2018; Ramirez-Campillo et al., 2023a) and a limited understanding on how well the training stimulus elicits an appropriate adaptation while assessing the influence of external and internal moderating variables (Moran et al., 2018; Ramirez-Campillo et al., 2023a). Therefore, the aims of this study are to explore the effect of horizontal plyometric training on SSC jumping and hopping and to assess the influence of training, age, body size and tissue related moderators on plyometric training in post-PHV girls. The first hypothesis of the study was that an 8-week plyometric training program with horizontal exercises would improve variables associated with stretch-shortening cycle function. The second hypothesis was that changes in variables associated with the stretch-shortening cycle would be moderated by anthropometric, functional, and training related variables such as body mass, total GJH, and training volume respectively.

## 2 Materials and methods

### 2.1 Participants

A total of 23 post-PHV female volleyball players between the ages of 11.8 and 15.8 years were selected to participate in this study. Local volleyball clubs in Olomouc, Czechia, were approached to ascertain their interest in participating in the research study, of which the volleyball academy VAM Olomouc agreed to participate. Afterwards the coaches approached the parents and their children to gauge their interest in participating. Ultimately most of the children and parents of VAM Olomouc agreed to participate. Inclusion criteria for initial participation included: a) participation in highest national youth level competition in the competitive age categories U13–U16; b) being free from major orthopedic injuries (e.g., sprains, fractures, and tears) for at least 3 months prior to the start of the study; c) being free from any pain which would limit safe participation. Only participants who completed both the pre-test and post-test component of a given assessment were taken forward for analysis. These participants were also required to attend at least 75% of all training sessions (Lloyd et al., 2012a). These criteria for analysis resulted in 28 participants being excluded. All participants and parents/guardians were informed of the benefits and risks of being a part of this study. Written parental consent and written participant assent were obtained prior to commencing all data collection procedures. All data collection procedures were reviewed and approved by the Auckland University of Technology Ethics Committee (reference number: 19/434) and the Ethics Committee of the Faculty of Physical Culture, Palacký University Olomouc (reference number: 15/2023). This study was conducted according to the Declaration of Helsinki regarding the use of human participants.

### 2.2 Study procedures

The current study, which used a quasi-experimental design, consisted of pre-testing and post-testing, separated by an 8-week intervention, with two sessions per week. Participants attended a testing familiarization session 1-week prior to the pre-testing sessions. Pre-testing consisted of two consecutive testing days. Day one consisted of a GJH test, a vertical bilateral sub-maximal hopping test, and a drop jump test, whereas day two consisted of a series of single and triple horizontal jumps and hops tests and vertical unilateral sub-maximal hopping tests. Pre-testing was conducted 1-week prior to the start of the intervention. For the post-testing, all tests were completed on 1 day, 3 days after the end of the intervention. During both pre-testing and post-testing, anthropometric measurements were taken. Participants were asked to refrain from any vigorous activity for 24 h prior to the testing sessions to limit the effects of fatigue (Turner et al., 2015). Each testing session began with a standardized warm-up which progressed from low intensity to high intensity, simple to complex and general to specific. The warm-up started with fundamental movement skills such as skipping, jogging, shuffling and dynamic stretching that targeted the calves, quadriceps, hamstrings and adductors. Following the fundamental movement skills and dynamic stretching, vertical bilateral and unilateral sub-maximal hopping, a series of single and triple horizontal jumps and hops of progressive intensity and three sprints of progressive intensity (60%, 90%, and 100% of maximal effort) were performed. Consistent verbal encouragement was used during all trials and sessions for each participant. All tests were performed inside the university sport facility. Tests were performed in the afternoon at the same time of the day by researchers qualified to deliver the testing and instruction.

### 2.3 Testing protocols

#### 2.3.1 Anthropometric and maturity measures

Standing height, seated height, body mass, body fat percentage, and hip width (bitrochanteric and biiliac) were measured. Stature measurements were completed using a portable stadiometer (Seca 213, Seca, Hamburg, Germany), after which leg length was determined by subtracting seated height from standing height. Bioelectric impedance analysis using the Tanita SC-240 (Tanita Corporation, Tokyo, Japan) was used for body mass and body fat percentage. Lastly, hip width at the level of the iliac crest and the level of the greater trochanter were measured using large bone calipers (Model 01293, Lafayette Instrument, Lafayette, IN, United States). Maturity timing was determined with a non-invasive method based on anthropometric variables, calendar age, date of birth, and testing date. This data was used in a regression equation to estimate maturity offset and age at PHV which indicate maturity timing (Mirwald et al., 2002). All maturity calculations were completed using the spreadsheets by Towlson et al. (2020).

#### 2.3.2 Sub-maximal hopping

Sub-maximal hopping data was collected instantaneously through a mobile contact mat with attached electronic hub (SmartJump. Fusion Sport, Brisbane, Australia). The calculation of leg stiffness using the method described by Dalleau et al. (2004) is valid and reliable in youth hopping on a contact mat (Lloyd et al., 2009). Absolute leg stiffness was determined by vertical bilateral hopping at a frequency of 2.0 Hz and 2.2 Hz. Conversely, leg stiffness was determined by vertical unilateral sub-maximal hopping at two different frequencies 2.5 Hz on the right leg, 2.5 Hz on the left leg, 3.0 Hz on the right leg, and 3.0 Hz on the left leg (Lloyd et al., 2009; Beerse and Wu, 2016). Hopping at 2.5 Hz has been referred to as the hopping frequency during which stiffness is best expressed (Beerse and Wu, 2016). Each participant completed one trial of 20 consecutive hops in which they attempted to match a frequency set by a digital metronome (Eiling et al., 2007; Lloyd et al., 2009; Lloyd et al., 2011b). To minimize fatigue, 3-min of passive rest were given between each hop type. Participants were instructed to keep their hands on hips, jump and land on the same spot, land with legs extended and maintain their gaze forwards to minimize the additive effect of the arms and trunk (Lloyd et al., 2009). The average stiffness of all contacts was used for analysis. Contacts in which the participant jumped off the mat then back or where participants misheard instructions and stopped for too long or lost rhythm (i.e., contacts exceeded 300 ms) were excluded.

#### 2.3.3 Horizontal jump and hop tests

A series of single and triple horizontal jump and hop tests were measured to the nearest 0.2 cm using a standard fiberglass tape measure. Hopping tests are typically executed by taking off and landing on the same leg, whereas jumping tests are typically executed by taking off and landing on two legs (McGann et al., 2020). The hopping tests of the present study used a novel technique, where they used a one leg take-off but a final landing on two feet.

Overall, maximum jump distance was measured over two trials each with a 1-min rest between attempts and 2-min rest between jump or hop type. The average of the two trials was used for analysis (Myers et al., 2014). Jumps were considered a fault if the participant moved their foot upon landing, or if the participant put their hands on the ground to stabilize themselves upon the final landing. Hops used the same criteria in addition to being a fault if the free leg touched the ground prior to the final landing. All jumps and hops were completed with the final landing on two feet.

##### 2.3.3.1 Broad jumps

For the single and triple broad jump tests, participants started in a standing position with the toes of both feet behind the start line. Both the single and triple broad jump required the participant to start with a countermovement and jump horizontally as far as possible either once or three consecutive times without pause respectively (Ramirez-Campillo et al., 2015a; Delextrat et al., 2015).

##### 2.3.3.2 Broad hops

For the single and triple broad hop tests, participants started in a standing position with the toes of one foot behind the start line. During the single broad hop test, participants performed a maximal hop for distance, completing the landing on two feet. The triple broad hop test required participants to perform three maximal consecutive hops for distance without pausing between hops and landing from the last hop on two feet (Turner and Jeffreys, 2010; Hammami et al., 2016).

#### 2.3.4 Drop jump

Drop jump testing using a height of 30 cm was used to measure RSI with an Opto-jump Next system (Microgate, Bolzano, Italy) with 0.001 s accuracy. The rest interval between attempts was 30 s. Participants were instructed to place their hands on their hips, maintain their gaze forwards and step off the box towards the ground and rebound upwards. They were instructed to complete this action while getting off the ground as quickly as possible and jump as high as possible (Dalleau et al., 2004). Participants were also instructed to keep their legs extended during the flight phase of the jump and refrain from tucking their legs upwards or outwards. Trials in which the participants noticeably stepped down or noticeably jumped up from the box were not included and were asked to be repeated. Three trials were performed and the average of the two best results were used for further analysis (Sole et al., 2007). RSI was calculated as the ratio between jump height and contact time (Flanagan and Comyns, 2008; Lloyd et al., 2009). This method has been shown to be valid and reliable in youth athletes (Lloyd et al., 2009).

#### 2.3.5 Generalized joint hypermobility

The GJH was tested using the Beighton score which is a valid and reliable criterion used in diagnosing this condition (Remvig et al., 2007). The score consists of five components: passive dorsiflexion and hyperextension of the fifth metacarpal joints, passive apposition of the thumbs to the forearms, passive hyperextension of the elbows and knees, and active forward trunk flexion with knees fully extended. Note that the first four elements can be given a maximum score of two points because these are performed bilaterally (i.e., one point for each hypermobile joint), whereas the last element has a maximum score of 1 point. Thus, the total score ranges from 0 to 9 points, a higher score indicating the greater extent of joint hypermobility. The assessment was performed by an experienced physiotherapist and followed standard protocols employing a hand-held goniometer (Smits-Engelsman et al., 2011).

### 2.4 Training program

The training program consisted of various horizontal oriented plyometric exercises. These exercises were reactive in nature and involved an SSC action, meaning they required the participants to rebound off the ground and project their bodies in the horizontal direction (Lloyd et al., 2011b). The intervention ran twice a week for a period of 8 weeks. Each session commenced with a warm-up consisting of 5–7 min of problem-based movement activities, progressing from low to high intensity. The duration of each session was 35–45 min and session volume were tracked by distance (Ramirez-Campillo et al., 2023b). Intensity was determined based on the magnitude of eccentric loading of each exercise (Lloyd et al., 2011c; Meylan et al., 2012). Each exercise had three to six sets which required the participant to traverse 10–25 m. Rest between sets was one to 2 min. Exercises were progressed over the course of the 8-week intervention by exercise technique complexity (Talukdar et al., 2022), intensity, and volume (Ramirez-Campillo et al., 2023a). The training intervention was implemented by an experienced strength and conditioning coach certified by the National Strength and Conditioning Association. Exercise quality was carefully observed to ensure proper execution and limit the risk of injury. Researchers routinely confirmed with the participants whether or not an exercise produced any pain. There were no injuries that occurred as a result of the intervention. Specific intervention details are in Table 1.

**TABLE 1:** Training program details.

Category	Sets	Distance (m)	Intensity (Eccentric load)	Technique progressions	Reference
MDJLC	3–4	10–20	2–Low	Bilateral jump jump stick, Unilateral hop hop stick	Hewett et al. (1996)
MDAH	3–4	10–20	3–Moderate	Travelling forwards, backwards and laterally (Emphasize ankle dorsi-flexion)	Kong (2018); Lephart et al. (2005)
Power skipping	3–4	10–20	3–Moderate	Skipping with rhythm—Skipping for height—Skipping for distance	Faigenbaum et al. (2009)
Galloping	3–4	10–20	3–Moderate	Galloping with rhythm- Galloping for height- Galloping for distance	Konukman et al. (2008)
Broad jumping	4–6	10–15	2–Low	Broad jump with reset—Two “pump” broad jump with reset—Repeat broad jump	McCormick et al. (2016); Delextrat et al. (2015); Cochrane and Booker (2014)
Horizontal hopping	5–6/leg	10–20	4/5–Mod/High	Rhythm hops (100 bpm)—Hop over cones—Hop for distance	Witzke and Snow (2000); Lephart et al. (2005)
Bounding	4–6	15–20	5–Mod/High	Diagonal bounding—Straight bounding (short)—Straight bounding (long)	Witzke and Snow (2000); Kong (2018); Hewett et al. (1996)

MDJLC, multi-directional jump landing combination; MDAH, multi-directional ankle hops; Mod, moderate.

### 2.5 Statistical analysis

Statistical analyses were performed in MATLAB R2020a with Statistics Toolbox (MathWorks, Natick, MA, United States). Data were presented using arithmetic mean and standard deviation. Total GJH score was also presented using median and interquartile range. For all statistical tests, *p* < 0.05 was considered statistically significant. The normality of the data was evaluated using a Shapiro-Wilk test. Data were also plotted on a quantile-quantile plot and visually examined by a statistician. Change in the dependent variable during the training program was calculated as post-test minus pre-test (Δ = post–pre). A specialized spreadsheet (Hopkins, 2015) was used to obtain changes expressed as percentages. The statistical significance of the change was evaluated using a one-sample two-tailed *t*-test. In addition to statistical significance, effect size was also calculated. Cohen’s *d* was calculated as *d* = M_Δ_/SD_pre_, where M_Δ_ was the mean value of delta scores and SD_pre_ was calculated from the pre-test values (baseline). The following thresholds were used to interpret the magnitude of *d*: trivial 0.00–0.19, small 0.20–0.49, moderate 0.50–0.79, and large ≥0.80 (Cohen, 1988).

Multiple regression analysis was used to determine whether individual changes in the dependent variable could be moderated by calendar age, maturity offset, body height, body mass, total GJH score, and training volume. Only linear moderators without interactions were considered. In Wilkinson notation, the regression model can be written as follows: Δy ∼ 1 + calendar age + maturity offset + body height + body mass + total GJH score + training volume, where Δy is the change during the training program in the selected dependent variable. Effect size for each moderator was calculated using the eta squared statistic *η* ^2^ = SS_effect_/SS_total_, where SSeffect is the sum of squares associated with the moderator and SStotal is the total sum of squares (Fritz et al., 2012). The following thresholds were used to interpret *η* ^2^: trivial 0.000–0.009, small 0.010–0.059, moderate 0.060–0.139, and large ≥0.140 (Cohen, 1988). To evaluate the relationship between changes in the dependent variable and one selected moderator, Pearson’s correlation coefficient (*r*) was calculated. The following thresholds were used to interpret the magnitude of *r*: trivial 0.00–0.09, small 0.10–0.29, medium 0.30–0.49, and large ≥0.50 (Cohen, 1988).

Power analysis was performed using G*Power version 3.1.9.7 (Faul et al., 2007). The level for statistical significance was set at α = 0.05 and the power was set at 1-β = 0.80. Under the first hypothesis, a large effect size (*d* = 0.8) was considered for the paired two-tailed *t*-test. The required sample size resulted in 15 participants. Under the second hypothesis, a large correlation (*r* = 0.5) was considered for the Pearson’s correlation coefficient. The required sample size resulted in 26 participants. Thus, 26 participants were required to test both hypotheses.

## 3 Results

### 3.1 Data normality

The characteristics of the participants are shown in Table 2. The median total GJH score was 3 and the interquartile range was 3. Although the total GJH score was an ordinal scale ranging from 0 to 8, the Shapiro-Wilk test did not reject normality (*p* = 0.20, Table 2). Therefore, total GJH score was considered quasi-normal and used as a moderator in the regression analysis without any transformation. Normality was rejected for standing height (*p* = 0.013, Table 2). Upon examination of the quantile-quantile plot, it was found that the non-normality was due to one player whose standing height was 187.4 cm. This value was not considered as outlier and this player was retained in further statistical analysis. The remaining variables used as moderators in the regression analysis had normal distributions (all *p* ≥ 0.15, Table 2).

**TABLE 2:** Characteristics of the participants (*n* = 23).

Variable	Mean ± SD	*p*
Chronological age (years)	13.8 ± 1.2	0.40
Maturity offset (years)	1.7 ± 0.8	0.15
Mirwald APHV (years)	12.1 ± 0.6	0.27
Standing height (cm)	165.4 ± 6.8	0.013
Seated height (cm)	84.5 ± 3.3	0.22
Leg length (cm)	81.0 ± 4.8	0.36
Body mass (kg)	58.1 ± 9.6	0.20
Body fat (%)	24.3 ± 5.8	0.56
Total GJH score	3.5 ± 2.1	0.20
Attended sessions	13.8 ± 1.2	0.014
Training volume (km)	6.20 ± 1.00	0.16

SD, standard deviation; *p*, statistical significance of Shapiro-Wilk normality test; APHV, age at peak height velocity; GJH, generalized joint hypermobility.

The results of testing the normality of the differences between pre-values and post-values are provided in Table 3. The quantile-quantile plot was visually inspected for 3 out of the total 18 variables for which normality was rejected according to the Shapiro-Wilk test (*p* < 0.05). Upon examination, the deviation from normality was assessed as acceptable and parametric statistical methods were used as they are considered robust for such deviations from normality (Ghasemi and Zahediasl, 2012).

**TABLE 3:** The effect of the plyometric training program on the dependent variables.

Variable	Pre-test	Post-test	Δ = post-pre	*p*_sw_	*p*_tt_	*d*
	Mean ± SD	Mean ± SD	Absolute (%)			
Standing height (cm)	165.4 ± 6.8	165.9 ± 7.0	0.5 (0.3%)	0.34	0.001	0.07
Seated height (cm)	84.5 ± 3.3	84.7 ± 3.3	0.2 (0.3%)	0.96	0.31	0.07
Leg length (cm)	81.0 ± 4.8	81.2 ± 5.0	0.2 (0.3%)	0.34	0.31	0.05
Body mass (kg)	58.1 ± 9.6	59.0 ± 10.1	1.0 (1.6%)	0.13	0.001	0.10
Body fat (%)	24.3 ± 5.8	24.2 ± 5.9	−0.1 (−0.4%)	0.40	0.68	−0.02
Sub-max hop 2.0 Hz (kN/m)	19.4 ± 4.0	21.4 ± 4.9	2.0 (9.5%)	0.67	0.021	0.50
Sub-max hop 2.2 Hz (kN/m)	23.2 ± 4.4	24.9 ± 4.8	1.6 (6.8%)	0.85	0.049	0.37
Sub-max hop 2.5 Hz right (kN/m)	19.0 ± 2.4	19.5 ± 2.7	0.5 (2.3%)	0.032	0.28	0.19
Sub-max hop 2.5 Hz left (kN/m)	18.9 ± 2.7	19.7 ± 3.0	0.9 (4.4%)	0.049	0.032	0.32
Sub-max hop 3.0 Hz right (kN/m)	23.7 ± 4.0	25.4 ± 4.7	1.6 (6.8%)	0.35	0.056	0.40
Sub-max hop 3.0 Hz left (kN/m)	24.1 ± 3.4	24.9 ± 4.0	0.8 (3.0%)	0.51	0.11	0.23
Broad jump (cm)	192 ± 21	187 ± 18	−5 (−2.4%)	0.84	0.012	−0.23
Broad hop right (cm)	164 ± 16	166 ± 13	2 (1.6%)	0.80	0.28	0.15
Broad hop left (cm)	168 ± 17	170 ± 14	3 (1.7%)	0.024	0.22	0.15
Triple broad jump (cm)	579 ± 58	569 ± 53	−10 (−1.6%)	0.34	0.13	−0.17
Triple broad hop right (cm)	493 ± 59	496 ± 50	3 (0.7%)	0.90	0.75	0.04
Triple broad hop left (cm)	491 ± 55	501 ± 60	10 (2.0%)	>0.99	0.18	0.18
Reactive strength index (m/s)	1.03 ± 0.31	1.04 ± 0.27	0.01 (2.0%)	0.84	0.66	0.04

SD, standard deviation; *p*_sw_, statistical significance of Shapiro-Wilk normality test; *p*_tt_, statistical significance of paired *t*-test; *d*, Cohen’s effect size; Sub-max, sub-maximal; Hz, Hertz.

### 3.2 Training effect

The effect of the plyometric training program on the examined dependent variables is shown in Table 3. After plyometric training, there was a statistically significant increase in standing height (pre: 165.4 ± 6.8, post: 165.9 ± 7.0 cm, *p* = 0.001, *d* = 0.07, trivial effect) and body mass (pre: 58.1 ± 9.6, post: 59.0 ± 10.1 kg, *p* = 0.001, *d* = 0.10, trivial effect), which was the expected growth effect. Importantly, the effect of training on body fat (pre: 24.3 ± 5.8, post: 24.2% ± 5.9%, *p* = 0.68, *d* = −0.02, trivial effect) was not significant. Among the vertical sub-maximal hopping variables, significant increases were found for vertical bilateral hopping at 2.0 Hz (pre: 19.4 ± 4.0, post: 21.4 ± 4.9 kN/m, *p* = 0.021, *d* = 0.50, medium effect), vertical bilateral hopping at 2.2 Hz (pre: 23.2 ± 4.4, post: 24.9 ± 4.8 kN/m, *p* = 0.049, *d* = 0.37, small effect), and vertical unilateral hopping at 2.5 Hz left (pre: 18.9 ± 2.7, post: 19.7 ± 3.0 kN/m, *p* = 0.032, *d* = 0.32, small effect). Examination of the broad jump variable revealed a significant decrease in broad jump distance (pre: 192 ± 21, post: 187 ± 18 cm, *p* = 0.012, *d* = −0.23, small effect).

### 3.3 Effect of moderators

The results of the regression analysis are presented in Tables 4–6. Table 4 contains the values of each regression coefficient, Table 5 contains the statistical significance for each regression coefficient, and Table 6 contains the eta-squared for each regression coefficient. Body mass moderated the following three dependent variables: vertical unilateral hopping at 2.5 Hz right (*p* = 0.010, *η* ^2^ = 0.314, large effect), vertical unilateral hopping at 3.0 Hz right (*p* = 0.035, *η* ^2^ = 0.170, large effect), and vertical unilateral hopping at 3.0 Hz left (*p* = 0.043, *η* ^2^ = 0.203, large effect). Additionally, total GJH score moderated both the triple broad hop right (*p* = 0.012, *η* ^2^ = 0.210, large effect) and the triple broad hop left (*p* = 0.034, *η* ^2^ = 0.226, large effect). Training volume significantly moderated only triple broad hop right (*p* = 0.024, *η* ^2^ = 0.160, large effect). Calendar age (all *p* ≥ 0.11), maturity offset (all *p* ≥ 0.12), and body height (all *p* ≥ 0.077) did not significantly moderate any dependent variable.

**TABLE 4:** Values of regression coefficients.

Variable	Calendar age	Maturity offset	Body height	Body mass	Total GJH score	Training volume
	[(y)/year]	[(y)/year]	[(y)/cm]	[(y)/kg]	[(y)/point]	[(y)/km]
Body mass (kg)	1.610	−3.153	0.184	0.054	0.220	−0.220
Body fat (%)	2.218	−4.338	0.204	0.046	0.197	0.376
Sub-max hop 2.0 Hz (kN/m)	−1.932	3.629	−0.333	0.060	−0.260	−1.719
Sub-max hop 2.2 Hz (kN/m)	1.142	−3.131	−0.060	0.176	−0.092	−1.337
Sub-max hop 2.5 Hz right (kN/m)	1.216	−2.661	0.061	0.171	−0.031	0.065
Sub-max hop 2.5 Hz left (kN/m)	−0.636	0.710	−0.112	0.122	−0.086	−0.006
Sub-max hop 3.0 Hz right (kN/m)	5.877	−10.151	0.648	0.245	0.211	−0.045
Sub-max hop 3.0 Hz left (kN/m)	0.135	−0.114	−0.051	0.162	−0.083	−0.025
Broad jump (cm)	−1.839	3.973	0.027	−0.064	−1.167	3.253
Broad hop right (cm)	−9.497	17.881	−0.597	−0.300	−2.337	3.673
Broad hop left (cm)	1.324	−5.075	0.869	−0.305	−1.966	3.067
Triple broad jump (cm)	−13.983	21.429	−1.146	0.249	−4.893	4.579
Triple broad hop right (cm)	−34.424	44.255	0.213	−0.464	−8.951	17.375
Triple broad hop left (cm)	−15.683	24.076	0.211	−0.494	−8.531	11.097
Reactive strength index (m/s)	−0.151	0.238	−0.010	−0.003	−0.022	0.010

GJH, generalized joint hypermobility; (y), unit of the dependent variable; Sub-max, sub-maximal; Hz, Hertz.

**TABLE 5:** Statistical significances of regression coefficients.

Variable	Calendar age	Maturity offset	Body height	Body mass	Total GJH score	Training volume
Body mass (kg)	0.18	0.15	0.12	0.14	0.078	0.41
Body fat (%)	0.16	0.13	0.19	0.33	0.21	0.28
Sub-max hop 2.0 Hz (kN/m)	0.65	0.63	0.42	0.64	0.54	0.082
Sub-max hop 2.2 Hz (kN/m)	0.79	0.68	0.89	0.19	0.83	0.18
Sub-max hop 2.5 Hz right (kN/m)	0.54	0.45	0.75	0.010	0.88	0.88
Sub-max hop 2.5 Hz left (kN/m)	0.74	0.84	0.56	0.050	0.66	0.99
Sub-max hop 3.0 Hz right (kN/m)	0.11	0.12	0.077	0.035	0.56	0.96
Sub-max hop 3.0 Hz left (kN/m)	0.96	0.98	0.83	0.043	0.74	0.96
Broad jump (cm)	0.85	0.82	0.98	0.83	0.25	0.15
Broad hop right (cm)	0.41	0.39	0.60	0.39	0.057	0.17
Broad hop left (cm)	0.89	0.77	0.36	0.30	0.056	0.17
Triple broad jump (cm)	0.70	0.74	0.74	0.82	0.19	0.57
Triple broad hop right (cm)	0.28	0.44	0.94	0.63	0.012	0.024
Triple broad hop left (cm)	0.67	0.72	0.95	0.66	0.034	0.19
Reactive strength index (m/s)	0.31	0.37	0.49	0.52	0.14	0.76

GJH, generalized joint hypermobility; Sub-max, sub-maximal; Hz, Hertz.

**TABLE 6:** Eta-squared of regression coefficients.

Variable	Calendar age	Maturity offset	Body height	Body mass	Total GJH score	Training volume
Body mass (kg)	0.075	0.090	0.102	0.091	0.136	0.028
Body fat (%)	0.093	0.111	0.082	0.044	0.072	0.053
Sub-max hop 2.0 Hz (kN/m)	0.010	0.011	0.031	0.010	0.017	0.154
Sub-max hop 2.2 Hz (kN/m)	0.003	0.008	0.001	0.091	0.002	0.095
Sub-max hop 2.5 Hz right (kN/m)	0.015	0.022	0.004	0.314	0.001	0.001
Sub-max hop 2.5 Hz left (kN/m)	0.005	0.002	0.016	0.193	0.009	0.000
Sub-max hop 3.0 Hz right (kN/m)	0.090	0.084	0.114	0.170	0.011	0.000
Sub-max hop 3.0 Hz left (kN/m)	0.000	0.000	0.002	0.203	0.005	0.000
Broad jump (cm)	0.002	0.003	0.000	0.002	0.073	0.114
Broad hop right (cm)	0.031	0.035	0.013	0.034	0.187	0.093
Broad hop left (cm)	0.001	0.003	0.032	0.042	0.156	0.077
Triple broad jump (cm)	0.009	0.006	0.006	0.003	0.104	0.018
Triple broad hop right (cm)	0.032	0.016	0.000	0.006	0.210	0.160
Triple broad hop left (cm)	0.008	0.006	0.000	0.008	0.226	0.077
Reactive strength index (m/s)	0.058	0.044	0.025	0.022	0.122	0.005

GJH, generalized joint hypermobility; Sub-max, sub-maximal; Hz, Hertz.

**FIGURE 1:**
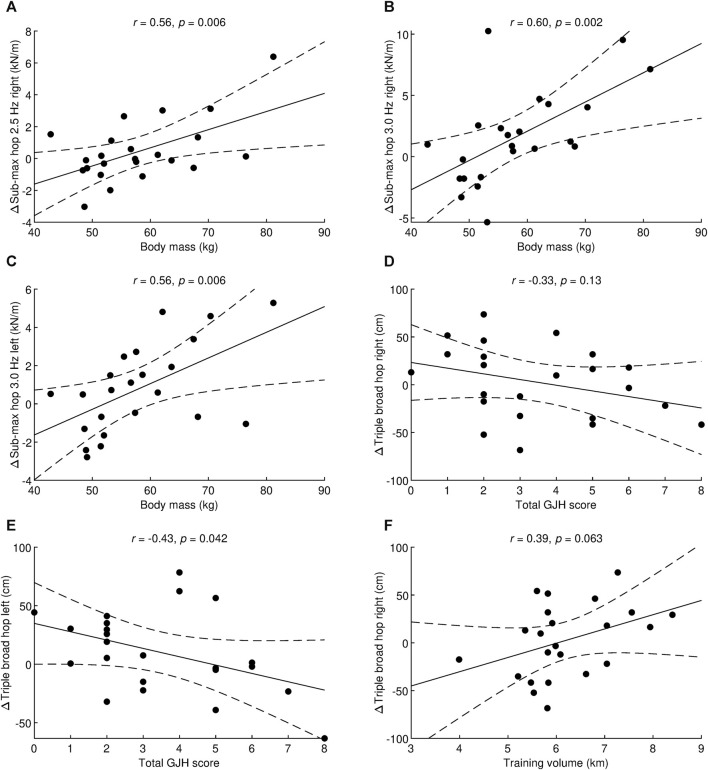
Correlation analysis between change in unilateral sub-maximal hopping at 2.5 Hz right and body mass **(A)**, change in unilateral sub-maximal hopping at 3.0 Hz right and body mass **(B)**, change in unilateral sub-maximal hopping at 3.0 Hz left and body mass **(C)**, change in triple broad hop right and total generalized joint hypermobility score **(D)**, change in triple broad hop left and total generalized joint hypermobility score **(E)**, and change in triple broad hop right and training volume **(F)**. *r*, Pearson’s correlation coefficient; *p*, statistical significance of the correlation coefficient; Δ, difference between post-test versus pre-test; Sub-max, sub-maximal; Hz, Hertz. Dashed lines denote a 95% confidence interval.

Interestingly, two simultaneous significant moderators were found for triple broad hop on the right leg, whereas the other dependent variables had at most one significant moderator. Therefore, a correlation analysis was performed to assess the association between changes in the dependent variable before and after the training program and one variable with pre-test values. Body mass alone was significantly correlated with the same three variables for which the regression analysis yielded a significant result mentioned above: vertical unilateral hopping at 2.5 Hz right (*r* = 0.56, *p* = 0.006, large effect, Figure 1A), vertical unilateral hopping at 3.0 Hz right (*r* = 0.60, *p* = 0.002, large effect, Figure 1B), and vertical unilateral hopping at 3.0 Hz left (*r* = 0.56, *p* = 0.006, large effect, Figure 1C). Total GJH score was significantly correlated only with triple broad hop left (*r* = −0.43, *p* = 0.042, medium effect, Figure 1E). Triple broad hop right was not significantly correlated with either total GJH score (*r* = −0.33, *p* = 0.13, Figure 1D) or training volume (*r* = 0.39, *p* = 0.063, Figure 1F). Thus, total GJH score and training volume together significantly influenced triple broad hop right, as shown in the regression analysis, but when these moderators were taken separately, no significant correlation was found.

## 4 Discussion

The primary aim of this study was to explore the effect of an 8-week horizontal plyometric training program on SSC jumping and hopping in post-PHV female volleyball players. The main findings of the current study demonstrated that an 8-week plyometric training program improved absolute leg stiffness at the preferred frequency (2.5 Hz, left leg) in post-PHV female volleyball players. To our knowledge this is the first intervention study to explore the effect of horizontal plyometric training on leg stiffness during vertical unilateral sub-maximal hopping and on explosive horizontal jumping and hopping ability using a novel protocol in post-PHV female volleyball players. The second aim was to assess the influence of training, age, body size, and tissue related (GJH) moderating variables on plyometric training. Regarding the interaction of plyometric responses and moderating variables, body mass moderated the effect of plyometric training on the largest number of parameters, specifically, vertical sub-maximal hopping at 2.5 Hz on the right leg, and at 3.0 Hz on the left and right leg. This is the first study to assess the influence training, age, body size, and tissue related (GJH) variables on such plyometric training in post-PHV female volleyball players. The results of the present study indicate that a sufficient training stimulus was provided to improve leg stiffness at the preferred hopping frequency in youth, but not for horizontal jumping and hopping ability or RSI. Lastly, body mass and not age, maturity, training volume or GJH served as the best moderator of horizontal plyometric training.

### 4.1 Training effect

The intervention in the current study consisted of horizontally oriented plyometric exercises that involved an SSC and were reactive in nature. The ability to rebound off the ground can be aided by leg stiffness, a component of effective SSC function (Radnor et al., 2017). Leg stiffness describes the ability to attenuate an applied force during deformation of the leg, where an optimal level of stiffness allows a large force to be attenuated over a shorter range of motion (McMahon and George, 1990). This training intervention led to a significant increase in absolute leg stiffness during vertical bilateral hopping at 2.0 Hz, 2.2 Hz, and vertical unilateral hopping at 2.5 Hz on the left leg. The medium intervention effect for sub-maximal hopping at 2.0 Hz (9.5%) and 2.5 Hz (4.4%) on the left leg exceeds the within subject error (2.0 Hz: 8.23%, 2.5 Hz: 3.89%) of a unpublished PhD thesis reliability study, and can thus be considered clinically significant (Sylvester, 2024). Furthermore, the small degree of intervention effect for sub-maximal hopping at 2.2 Hz (6.8%) did not exceed the within subject error (7.25%) of a unpublished PhD thesis reliability study and are not considered clinically significant (Sylvester, 2024). Furthermore, the significant improvement in leg stiffness during vertical unilateral hopping at 2.5 Hz on the left and not the right leg agrees with the unpublished PhD thesis reliability study (Sylvester, 2024), during which the left leg represented the non-dominant leg for most participants, as determined by the kicking leg. The increased reliability of left leg vertical hopping might have influenced why significant results were demonstrated on that leg in the current investigation.

Changes larger than the within subject error indicate that plyometric training can induce changes in leg stiffness over and above what would be expected by natural development (Radnor et al., 2017). The findings of the present study are relevant in demonstrating that a horizontal plyometric stimulus can also lead to improvement in vertical outcomes other than vertical countermovement jumps in post-PHV female athletes (Talukdar et al., 2022), in this case, absolute leg stiffness during unilateral vertical sub-maximal hopping. In addition to body mass and maturity, muscle pre-activation and the stretch-reflex response of the leg extensors, are the major predictors of absolute leg stiffness in youth, explaining approximately 97% of the variance in leg stiffness (Oliver and Smith, 2010). In the present study, where maturity did not moderate change in leg stiffness and body mass did, increased leg stiffness observed in youth female volleyball players after the plyometric training program could also presumably be explained by muscle activity (Oliver and Smith, 2010).

The current study suggests that post-PHV female volleyball players can improve leg stiffness after a plyometric training program. However, the effect of plyometric training on leg stiffness in post-PHV female athletes overall still requires further investigation. Moreover, the effect of plyometric training on leg stiffness in pre-PHV or circa-PHV girls also remains unclear due to a paucity of studies. It is for this reason studies in boys are used to help elucidate these effects. For example, improvement in absolute and relative leg stiffness in 12- and 15-year-old male physical education students have been demonstrated during vertical bilateral hopping at 2.5 Hz after a 4-week plyometric training program. The same study revealed that 9-year-old physical education students did not demonstrate improvements in leg stiffness (Lloyd et al., 2012a). Similar investigations have not been conducted in girls, therefore, further studies in youth female athletes are needed to affirm the effect of plyometric training on leg stiffness in general. Moreover, it is desirable to ascertain the effect of plyometric training on leg stiffness in youth female athletes across maturation in comparison to a control group to further discern the training effect from the natural development effect.

The present study did not reveal significant increases in RSI after a plyometric training intervention. This finding indicates that while potential benefits for SSC behavior could be expected due to increased leg stiffness, other aspects of SSC performance were not improved. More specifically, the RSI parameter in our study derived from the drop jump test, is used to assess an athlete’s ability to produce force rapidly. Higher values of the RSI are associated with the effective use of muscle elasticity and neuromuscular control of working muscles (Flanagan and Comyns, 2008; Jarvis et al., 2022). Observation of the discrepancy between training induced effects in absolute leg stiffness and RSI indirectly supports a previous suggestion, that RSI has only a limited amount of variance with leg stiffness (Lloyd et al., 2011a). Absence of benefit in the case of RSI agrees with previous studies in 8-year-old female gymnasts after an 8-week plyometric training intervention (Bogdanis et al., 2019), and in 8-year-old female gymnasts after a combined 8-week plyometric, muscular strength, muscular endurance, movement competency and dynamic stabilization training program (Moeskops et al., 2018). However, our finding regarding RSI is not commensurate with findings of a recent systematic review with meta-analysis by Ramirez-Campillo et al. (2023a), which revealed significant gains in RSI after plyometric training programs in males and females under 18 years old. This systematic review with meta-analysis by Ramirez-Campillo et al. (2023a) also did not find sex or maturational status to be a significant moderator of plyometric training effect on RSI. Moreover, another recent systematic review with meta-analysis on healthy individuals across the lifespan revealed that plyometric jump training was effective (ES = 0.54, small effect) at improving leg stiffness (Ramirez-Campillo et al., 2023b). We assume that one of the reasons why positive changes of the RSI were not observed in our study were the differences between movement content of the current investigation’s horizontal plyometric program exercises, and the vertical nature of the drop jump test. As it is generally accepted that RSI is an important measure of SSC capability in youth athletes related to both athletic performance (Flanagan and Comyns, 2008; Ramirez-Campillo et al., 2018; Jarvis et al., 2022) and injury prevention (Toumi et al., 2006; Raschner et al., 2012) and considering that the natural development of RSI differs in boys and girls (Laffaye et al., 2016; De Ste Croix et al., 2021; Lehnert et al., 2022), existing ambiguities concerning potential benefits of plyometric training on RSI in youth female athletes should be clarified. Therefore, more investigations into different lengths and types of plyometric training interventions in girls are required to elucidate this theory. It must also be considered that the sport and training experience level and sex of participants are important, given that improvements may be more difficult to induce in girls or more athletically experienced individuals (Faigenbaum et al., 2002; Asadi et al., 2017; Moran et al., 2018; Moeskops et al., 2022b; Lehnert et al., 2022) and that in youth (age <18 years) smaller training induced changes can be expected compared to adults (Ramirez-Campillo et al., 2023b).

Horizontal plyometric training did not induce a significant improvement in any of the horizontal jumps or hops. Despite the 2.4% decrease in the broad jump, this decrease was not considered significant, given that it did not exceed the previously stated within subject error of 2.82% from a unpublished PhD thesis (Sylvester, 2024). It is difficult to explain with certainty as to why there was no significant improvement in horizontal jump and hop movements. One explanation is that horizontally directed plyometric exercises require the body to be projected into the air and across the ground, which may require a higher level of coordination to execute (Ramirez-Campillo et al., 2015b). This might be especially true when considering the reality of teaching exercises to young athletes. Horizontally directed plyometric exercises require a higher level of coordination compared to vertically oriented plyometric exercises (Fukashiro et al., 2005; Ramirez-Campillo et al., 2023a). Given that intensity is a vital consideration for plyometric training effectiveness (Brauner et al., 2014; Maloney et al., 2015; Ramirez-Campillo et al., 2023a), it is possible that while learning to coordinate these movements, the ability to perform these exercises maximally was affected.

Significant improvements in standing long jump (broad jump) have been demonstrated in girls from various sports and similar age range as players in the current study (age 13–16). For example, after a 7-week horizontal jump training intervention (Talukdar et al., 2022), after both 8-week bilateral and unilateral plyometric interventions (Kong, 2018), after both sagittal plane and frontal plane 6-week plyometric interventions (McCormick et al., 2016), after a 10-month combined movement competency, strength, ballistic, plyometric and speed training program (Moeskops et al., 2022a), and after an 8-week plyometric, speed and strength training intervention (Marta et al., 2014). The disagreement with the literature also applies to horizontal single and triple hopping where 15-year-old soccer, basketball and volleyball players were able to significantly improve their single leg hop distance after a 6-week neuromuscular training program (Myer et al., 2005). Similarly, 13–18-year-old athletic girls from various sports significantly improved in single leg triple hop distance, after a 6-week combined, jumping, plyometric, speed, endurance, agility training program (Noyes and Barber–Westin, 2015). However, differences between the current study and those in the literature are noted for movement orientation, analysis method (McCormick et al., 2016; Kong, 2018), intervention length (McCormick et al., 2016; Moeskops et al., 2022a), training content (Marta et al., 2014; Moeskops et al., 2022a), and sport background (Marta et al., 2014; McCormick et al., 2016; Moeskops et al., 2022b). These methodological differences revealed throughout the literature make clarifying the effect of plyometric training on the broad jump test difficult. The existence of the external moderators noted does not allow for clarity as to the effect of plyometric training on horizontal jumping and hopping in youth female athletes. This uncertainty is confirmed due to the findings of a recent systematic review and meta-analysis, where small significant effect sizes (d = 0.42–0.56) were found for the effect of plyometric training on horizontal jump distance (Ramirez-Campillo et al., 2023a).

### 4.2 Effect of moderators

The adaptive response to plyometric training may be influenced by various moderators (Moran et al., 2018; Ramirez-Campillo et al., 2023a). The present study sought to determine the influence of several moderators on plyometric training. However, the present study discovered that only body mass and total GJH combined with training volume had a moderating effect on plyometric training in post-PHV female volleyball players. Therefore, there remains uncertainty regarding the effect of moderators on plyometric training in girls.

The present study discovered that in a sample of post-PHV volleyball girls, there were no moderating effects of age, height or maturity offset. These findings agree with a meta-analysis by Moran et al. (2018) who demonstrated that older, taller and heavier girls adapted to plyometric training to a lesser extent compared to younger, lighter and shorter girls. In the study by Moran et al. (2018), which used a median split analysis, girls above age 15 were considered older. The girls in the present study were 13.8 years old, initially suggesting they should share similar characteristics (similar moderating effect of age on plyometric training) to the girls below the median in the study by Moran et al. (2018). However, this was not the case. Furthermore, when compared to girls in Moran et al. (2018), the girls of the present study were taller than the median height of the participants in Moran et al. (2018). This would suggest they resemble the older girls of Moran et al. (2018) and thus the reason for the lack of moderating effect of height or age in the present study.

Chronological age can be misleading as a moderator variable, given that many changes during adolescence occur due to increased biological age (Lloyd et al., 2016). However, in the study by Moran et al. (2018), there was no analysis completed regarding the moderating effect of maturation on plyometric training (Moran et al., 2018). This is not surprising given the scarcity of studies in girls where researchers have assessed maturation during plyometric training interventions. Currently there is no consensus regarding how girls of different maturity status adapt to plyometric training. For instance, in a recent systematic review with meta-analysis, it was shown that there is a paucity of studies in young girls where the moderating effect of maturation has been assessed during plyometric training interventions (Ramirez-Campillo et al., 2023a). Additional studies are required in youth female athletes where researchers assess maturation so a consensus can be generated regarding the moderating effect of maturation on plyometric training. It is also suggested that additional moderator analysis, other than anthropometric variables, be investigated by researchers which assess the functional qualities of the neuromuscular and musculoskeletal systems (Beerse and Wu, 2022).

#### 4.2.1 Effect of training volume

The present study revealed that training volume moderated one dependent variable but only when combined with the moderating effect of total GJH score. Specifically, a higher training volume and a lower total GJH score led to a higher value of triple broad hop on the right leg. The absence of significant effect of training volume may be attributed to the attendance criteria of 75% or more. This criterion limited the variability in the training volume across the participants (Lloyd et al., 2012a).

In the present study, training volume did not significantly moderate the effect of plyometric training on RSI. This finding is in line with findings of a previous systematic review with meta-analysis demonstrating that training volume in the form of number of weeks, total jumps, and total training sessions did not significantly moderate the effect of plyometric training on RSI (Ramirez-Campillo et al., 2023a) and vertical countermovement jumps in youth under 18 years old (Ramirez-Campillo et al., 2023a). However, in another recent systematic review with meta-analysis, the authors noted that three sessions per week, more than 7 weeks and more than 14 sessions, was a more effective dose to improve RSI in healthy people across the lifespan than under three weekly sessions, under 14 total sessions and under 14-week of training (Ramirez-Campillo et al., 2023b). Therefore, there may be differences between how youth under 18 years old respond to plyometric training compared to individuals across the lifespan.

Despite the significant increase in absolute leg stiffness during bilateral hopping at 2.0 Hz and left leg hopping at 2.5 Hz, training volume did not significantly moderate the effect of plyometric training on absolute leg stiffness. Stiffness in healthy individuals across the lifespan was shown to increase due to lower plyometric training volume, specifically, under 16 sessions, under three sets, and equal to or under 2 sessions per week (Moran et al., 2023). This relationship between training and stiffness differs from that of training volume and RSI for healthy individuals across the lifespan. It is likely that different expressions of the SSC like stiffness and RSI, have dissimilar dose-adaptation relationships. More studies investigating the effects of training volume with different numbers of jumps, weekly frequency, total sessions, and number of training weeks in the context of growth and maturation are required to clarify this idea.

#### 4.2.2 Effect of body mass

Correlation analysis was used to unveil the relationship between changes in each dependent variable and body mass at pre-test. Change in absolute leg stiffness during right leg vertical sub-maximal hopping at 2.5 Hz (*r* = 0.56) and 3.0 Hz (*r* = 0.60) and left leg vertical hopping at 3.0 Hz (*r* = 0.56) were correlated to body mass at pre-test. These findings indicate that when evaluating the effect of plyometric training on leg stiffness at preferred vertical hopping frequencies (2.5 Hz) and faster hopping frequencies (3.0 Hz), increased body mass at pre-test moderates plyometric training to produce higher leg stiffness. During the reactive hops, the entire body mass must be projected into the air against gravity after rebounding off the ground. This rebounding process is aided by increased amounts of leg stiffness, which helps attenuate large ground reaction forces without a large deformation (Lloyd et al., 2009; Pedley et al., 2020). Specifically, if leg stiffness does not increase with a larger mass, then the body will display a diminished ability to consistently control the landing forces and maintain an appropriate level of deformation to meet athletic task demands (Lloyd et al., 2009; Meyers et al., 2016; Pedley et al., 2020).

Athletic tasks executed in sport impose mechanical loading which leads to stiffness changes in youth (Waugh et al., 2012). This training and sport related loading can also be accompanied by chronic mechanical loading from growth and maturation related body mass increases, which also lead to increased stiffness in young people (Waugh et al., 2012; Chalatziglidis et al., 2021). This notion is supported by literature demonstrating that body mass is a primary contributor to absolute leg stiffness in 11–12-year-old boys, explaining from 61.7% to 75.8% of the variation during vertical hopping at preferred frequencies (Oliver and Smith, 2010; Lloyd et al., 2011a). Other significant contributors include extensor muscle activity upon ground contact (Oliver and Smith, 2010), which can be improved from training and sport related loading (Waugh et al., 2012). However, it is also known that girls increase their body fat mass to a higher degree compared to boys throughout childhood and adolescence (Naughton et al., 2000). This body composition change can negatively affect relative force production and impulse during reactive movements like jumping (Emmonds et al., 2017). This previous finding has been demonstrated in a meta-analysis in which heavier girls that were taller and older, adapted to plyometric training to a lesser degree compared to lighter, younger and shorter girls (Moran et al., 2018). Thus, to offset the negative effects of maturity related increases in fat mass, appropriate training, and sport related loading, like plyometric training and strength training, may help increase extensor muscle activity and thus stiffness (Ramirez-Campillo et al., 2023a; Moran et al., 2023). Previous studies in girls have not investigated the moderating effect of body mass on the change in performance due to a plyometric training program. There remains much variation unexplained in girls concerning SSC performance (Ramirez-Campillo et al., 2023a). Therefore, SSC jump and hop performance is not solely affected by body mass but is affected by maturation related neuromuscular factors that aid in controlling landing forces to help produce a forceful subsequent propulsive phase (Meylan et al., 2014).

#### 4.2.3 Effect of age and maturity

Correlation analysis did not reveal a significant relationship between the change in any of the performance variables and maturity offset or age. Nevertheless, it must be considered that the present study did not evaluate girls across maturation, and instead evaluated post-PHV girls only. The present study’s results thus indicate that for girls between 11.8 and 15.8, maturity timing and age did not significantly influence the effect of plyometric training on jumping and hopping ability. There is a previous meta-analysis that discovered that the effect of plyometric training on jumping performance was smaller among older (above 15 years), taller (above 163 cm) and heavier girls (above 54 kg) compared to younger, shorter and lighter girls (Moran et al., 2018). Moreover, in a systematic review with meta-analysis in healthy individuals across the lifespan, it was discovered that RSI improvements were better in adults compared to youth after plyometric training (Ramirez-Campillo et al., 2023b). However, the populations investigated in Moran et al. (2018) were girls of a similar and older age to the present study, whereas Ramirez-Campillo et al. (2023b) investigated healthy individuals across the lifespan. Direct comparisons between these previous studies and the present study are difficult. It has been suggested that the SSC develops non-linearly with age (Radnor et al., 2017), a finding which has been discovered for RSI in 8–13-year-old figure skating girls (Lehnert et al., 2022), 11–20-year-old girls (Laffaye et al., 2016), and 7–16-year-old boys (Lloyd et al., 2011b). Furthermore, leg stiffness in girls has also been shown to develop non-linearly with age although, the significant increases occurred within age ranges not commensurate with those of the present study (Laffaye et al., 2016). Given the lack of moderating effects of age and maturation, it could be that the majority of participants in the present study were of an age in which plyometric training is not moderated by age or maturation. Overall, this study suggests that plyometric training may be an effective method for improving leg stiffness in post-PHV female athletes, regardless of their age and maturity status. Further research is needed to confirm age and maturity ranges which influence jumping and hopping ability in girls. These studies should include pre-PHV participants and girls under 11 years old.

#### 4.2.4 Effect of hypermobility

The present study revealed that total GJH score moderated the triple broad hop right and the triple broad hop left. Subsequent correlation analysis was performed to determine the relationship between changes in the triple broad hops after the plyometric training program. The correlation analysis revealed that total GJH score only moderated the plyometric training effect on left leg triple broad hop performance, whereas total GJH score and training volume together significantly moderated the plyometric training effect on right leg triple broad hop performance. Given the negative correlations, this finding indicates that a lower total GJH score is associated with a higher ability to perform repetitive horizontal hops. Increased GJH may negatively affect force transmission and tissue elasticity, potentially leading to excessive tissue strain and joint excursions, thus causing a decreased ability to control landing forces through an appropriate deformation (Simmonds, 2022). Under these conditions, the ability to rebound effectively off the ground is diminished (Getchell and Roberton, 1989; Tveter and Holm, 2010). Comparisons to previous literature for this parameter are not possible since there are no studies that have investigated the moderating effect of total GJH score on plyometric training in girls. Thus, further investigations are required to clarify this moderating effect of total GJH score on plyometric training in girls, and the effect of total GJH score overall on physical qualities like speed, power, strength, and stiffness in girls.

#### 4.2.5 Limitations

The results of the present study must be interpreted with caution due to a few limitations. The first limitation was that many participants were excluded due to low attendance rates, or missing data. This removal resulted in a final sample size of 23, which was less than the required sample size of 26 obtained from the power analysis, reducing the statistical power to observe a true effect (Turner et al., 2015). The second limitation is that only post-PHV girls were evaluated. This resulted in unequal representation of participants of different chronological and biological ages. Therefore, we recommend replicating this study using pre-PHV participants and comparing them to post-PHV participants. Lastly, considering the high level of coordination required for horizontal movements and the practicality of teaching exercises to youth, it is our recommendation to give a longer familiarization period for horizontally directed plyometric exercises. The present investigation used a minimal dose of intervention exercises during the 1-week familiarization. Upon reflection, this period and dose is deemed insufficient to properly familiarize participants to horizontal plyometric exercises. Thus, 2 weeks of familiarization before executing an 8-week training period would presumably give a more appropriate plyometric training dose to elicit improvement in horizontal plyometric exercises and potentially RSI.

## 5 Conclusion

The present study concludes that 8 weeks of horizontal plyometric training can improve unilateral absolute leg stiffness in post-PHV female volleyball players, and this training effect can be moderated by body mass. Furthermore, the training effect on triple broad hopping performance on the right leg can be moderated by combined training volume with total generalized joint hypermobility. This finding suggests leg stiffness, reactive strength index, single and triple horizontal jumping and hopping may require different training dosages in post-PHV female volleyball players. Future recommendations include utilizing a longer familiarization period, a longer intervention period, and investigations that include pre-PHV participants.

## 6 Practical implications

It seems that an 8-week horizontal plyometric training intervention increases absolute leg stiffness in post-PHV female volleyball players and that out of the observed moderators, only initial body mass of the players may influence the training effect. Concerning training induced changes, it seems that increases in leg stiffness indicate positive changes from both a performance related and an injury-prevention related point of view. However, no other observed parameter was improved after the intervention. For practitioners, it must be stressed that the results of the present study should be used with caution as specific programming parameters (horizontal plyometric exercises and their progression, etc.), program duration and volume, were applied to a specific group of post-PHV female volleyball players. Moreover, results of the study, in some cases, contradict the findings regarding studies with girls of similar characteristics. Therefore, further investigation is needed in this field to provide practitioners with valid plyometric training recommendations.

## Data Availability

The raw data supporting the conclusion of this article will be made available by the authors, without undue reservation.
